# Paliperidone: 3-{2-[4-(6-fluoro-1,2-benzoxazol-3-yl)piperidin-1-yl]eth­yl}-9-hy­droxy-2-methyl-1,6,7,8,9,9a-hexa­hydro­pyrido[1,2-*a*]pyrimidin-4-one

**DOI:** 10.1107/S160053681104164X

**Published:** 2011-10-12

**Authors:** Richard Betz, Thomas Gerber, Eric Hosten, Alaloor S. Dayananda, Hemmige S. Yathirajan, Saji Thomas

**Affiliations:** a Nelson Mandela Metropolitan University, Summerstrand Campus, Department of Chemistry, University Way, Summerstrand, PO Box 77000, Port Elizabeth 6031, South Africa; b University of Mysore, Department of Studies in Chemistry, Manasagangotri, Mysore 570 006, India; c Jubilant Life Sciences Ltd, C-26, Sector 59, Noida 201 301, India

## Abstract

The title compound (also known as 9-hy­droxy­risperidone), C_23_H_27_FN_4_O_3_, is a heterocyclic compound with manifold pharmacological properties. The hy­droxy group shows disorder over two positions, with site-occupancy factors of 0.856 (2) and 0.144 (2). The piperidine ring adopts a chair conformation, while the annulated ring bearing the hy­droxy group is present in a half-chair conformation. Classical O—H⋯O hydrogen bonds as well as C—H⋯N contacts connect the mol­ecules into undulating sheets lying perpendicular to the crystallographic *b* axis. The shortest centroid–centroid distance between two centers of gravity is 3.5867 (8) Å and is apparent between the benzoxazole moiety and the six-membered ring bearing the keto substituent.

## Related literature

For pharmacological background, see: de Leon *et al.* (2010 ▶); Spina & Crupi (2011 ▶). For related structures, see: Peeters *et al.* (1993 ▶); Ravikumar *et al.* (2005 ▶); Sun & Zhang (2009 ▶); Wang & Pan (2006 ▶). For graph-set analysis of hydrogen bonds, see: Etter *et al.* (1990 ▶); Bernstein *et al.* (1995 ▶). For puckering analysis, see: Cremer & Pople (1975 ▶).
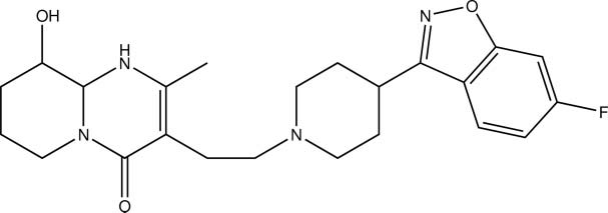


## Experimental

### 

#### Crystal data

C_23_H_27_FN_4_O_3_
*M* *_r_* = 426.49
Monoclinic, 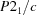

*a* = 6.8537 (1) Å
*b* = 21.5613 (5) Å
*c* = 15.3472 (3) Å
β = 113.857 (1)°
*V* = 2074.15 (7) Å^3^
*Z* = 4
Mo *K*α radiation
μ = 0.10 mm^−1^
*T* = 200 K
0.52 × 0.37 × 0.23 mm

#### Data collection

Bruker APEXII CCD diffractometer
Absorption correction: multi-scan (*SADABS*; Bruker, 2008 ▶) *T*_min_ = 0.944, *T*_max_ = 1.000
19267 measured reflections
5138 independent reflections
4130 reflections with *I* > 2σ(*I*)
*R*_int_ = 0.015

#### Refinement

*R*[*F* ^2^ > 2σ(*F* ^2^)] = 0.045
*wR*(*F* ^2^) = 0.130
*S* = 1.07
5138 reflections
291 parameters
3 restraints
H-atom parameters constrained
Δρ_max_ = 0.39 e Å^−3^
Δρ_min_ = −0.30 e Å^−3^

### 

Data collection: *APEX2* (Bruker, 2010 ▶); cell refinement: *SAINT* (Bruker, 2010 ▶); data reduction: *SAINT*; program(s) used to solve structure: *SHELXS97* (Sheldrick, 2008 ▶); program(s) used to refine structure: *SHELXL97* (Sheldrick, 2008 ▶); molecular graphics: *ORTEP-3* (Farrugia, 1997 ▶) and *Mercury* (Macrae *et al.*, 2008 ▶); software used to prepare material for publication: *SHELXL97* and *PLATON* (Spek, 2009 ▶).

## Supplementary Material

Crystal structure: contains datablock(s) I, global. DOI: 10.1107/S160053681104164X/zl2406sup1.cif

Supplementary material file. DOI: 10.1107/S160053681104164X/zl2406Isup2.cdx

Structure factors: contains datablock(s) I. DOI: 10.1107/S160053681104164X/zl2406Isup3.hkl

Supplementary material file. DOI: 10.1107/S160053681104164X/zl2406Isup4.cml

Additional supplementary materials:  crystallographic information; 3D view; checkCIF report

## Figures and Tables

**Table 1:** Hydrogen-bond geometry (Å, °)

*D*—H⋯*A*	*D*—H	H⋯*A*	*D*⋯*A*	*D*—H⋯*A*
O1—H1⋯O2^i^	0.84	1.86	2.6945 (16)	174
O1*B*—H1*B*⋯O2^i^	0.84	2.39	3.153 (8)	152
C4—H4*A*⋯N4^ii^	0.99	2.55	3.4830 (19)	157

Symmetry codes: (i) 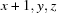; (ii) 
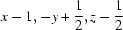
.

**Fig. 1.**
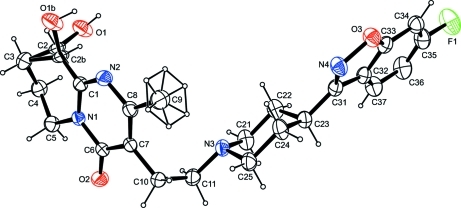
The molecular structure of the title compound, with atom labels and anisotropic displacement ellipsoids (drawn at 50% probability level).

**Fig. 2.**
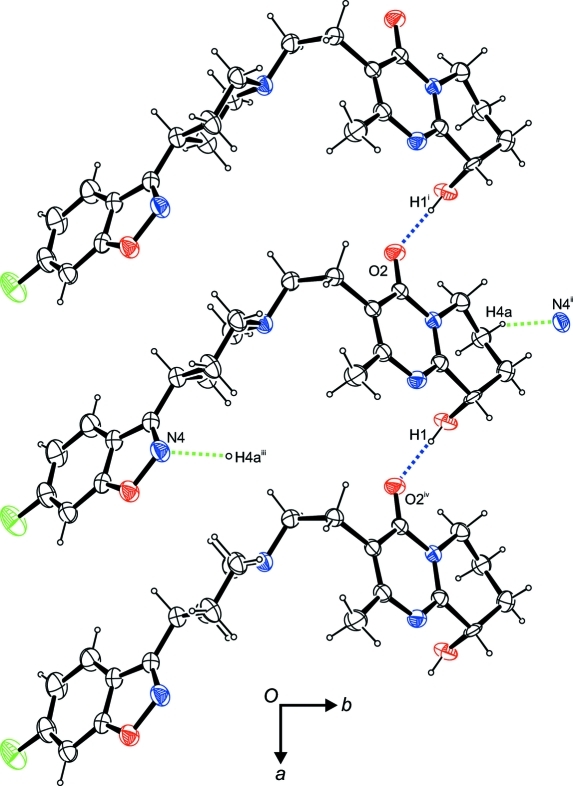
Selected intermolecular contacts, viewed along [0 0 - 1]. For clarity, only the main components of the disordered parts of the molecule are depicted. Blue dashed lines indicate classical hydrogen bonds of the O–H···O type, green dashed lines indicate C–H···N contacts. Symmetry operators: ix - 1, y, z; iix - 1, -y + 1/2, z - 1/2; iiix + 1, -y + 1/2, z + 1/2; ivx + 1, y, z.

**Fig. 3.**
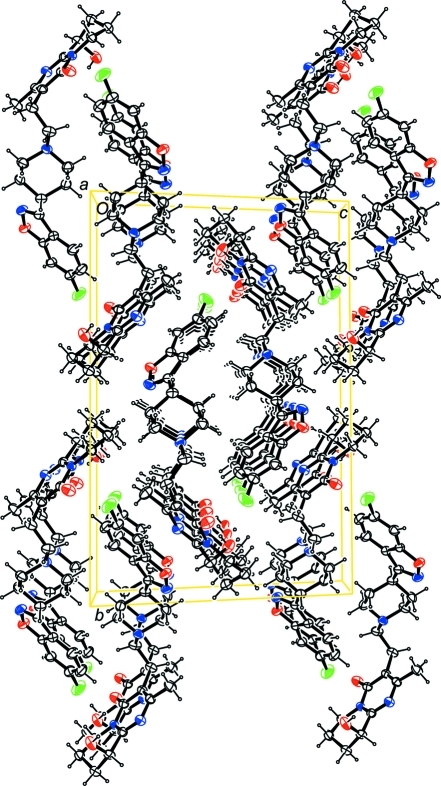
Molecular packing of the title compound, viewed along [-1 0 0] (anisotropic displacement ellipsoids drawn at 50% probability level). For clarity, only the main components of the disordered part of the molecule are depicted.

## supplementary crystallographic information

### Comment

Paliperidone, or 9-hydroxyrisperidone, is one of the most recently available atypical antipsychotics (Spina & Crupi, 2011). It is a benzisoxazole derivative and the major active metabolite of risperidone, a widely used atypical antipsychotic approved for the treatment of schizophrenia and other psychiatric disorders. The pharmacokinetics of paliperidone *versus* risperidone have been published (de Leon *et al.*, 2010). Related crystal structures, *viz.* 3-{2-[4-(6-fluoro-1,2-benzisoxazol-3-yl)piperidino]ethyl}-6,7,8,9-tetrahydro-\ 2-methyl-4*H*-pyrido[1,2-*a*]pyrimidin-4-one [risperidone] (Peeters *et al.*, 1993), risperidone *N*-oxide hydrogen peroxide methanol solvate (Ravikumar *et al.*, 2005), risperidone chloride 2.5 hydrate (Wang & Pan, 2006), 4-(6-fluoro-1,2-benzisoxazol-3-yl)-1-[2-(2-methyl-4-oxo- 6,7,8,9-tetrahydro-4*H*-pyrido[1,2-*a*] pyrimidin-3-yl)ethyl]piperidinium nitrate (Sun & Zhang, 2009) have been reported. In view of the importance of the title compound we herein report its molecular and crystal structure.

The hydroxy group as well as the hydrogen atoms of the methyl group show disorder. While the hydroxy group is disordered over two defined positions with site occupancy factors of of 0.856 (2) and 0.144 (2), rotational disorder is observed for the hydrogen atoms of the methyl group (occupancy ratio 0.68 (2) to 0.32 (2)). The low puckering amplitude of the six-membered ring bearing the keto group preculdes a conformational analysis (Cremer & Pople, 1975). The piperidine ring is present in a ^1^*C*_4_ conformation (^N3^*C*_C23_) and the hydroxy-tetrahydropyrido ring annulated on the pyrimidin-4-one ring adopts a ^5^*H*_4_ conformation (^C4^*H*_C3_) (Fig. 1). Proton NMR spectra of dissolved crystals of the title compound do not indicate the presence of the two stereoisomers as became apparent upon modelling the disorder for the hydroxy group in an axial-equatorial configuration.

In the crystal, classical hydrogen bonds of the O–H···O type as well as C–H···N contacts whose range falls by 0.2 Å below the sum of van-der-Waals radii can be observed. While the classical hydrogen bonds are apparent between the hydroxy group as donor and the keto group as acceptor, the C–H···N contacts appear between one of the methylene groups of the central aza-cyclohexane moiety and the nitrogen atom of the oxazol subunit. In terms of graph-set analysis (Etter *et al.*, 1990; Bernstein *et al.*, 1995), the descriptor for the classical hydrogen bonds is *C*^1^_1_(7) whereas the C–H···N contacts necessitate a *C*^1^_1_(14) descriptor on the same level. In total, the molecules are connected to undulated sheets perpendicular to the crystallographic *b* axis. The shortest intercentroid distance between two centers of gravity was found at 3.5871 (8) Å and is observed between the oxazol subunit and the six-membered heterocycle bearing the keto-group (Fig. 2). Furthermore, a F···*C*_g_ contact (*d*_F···_*_Cg_*: 3.2038 (12) Å) is observed between the fluorine atom and the six-membered ring bearing the keto group.

The packing of the title compound is shown in Figure 3.

### Experimental

The title compound was obtained as a gift sample from Jubilant Life Sciences Ltd., Noida, India. Paliperidone was recrystallized from *N*,*N*-dimethylformamide by slow evaporation at room temperature.

### Refinement

Four reflections, 1 0 4, -1 3 1, 0 4 4 and 0 1 1 were found to be obstructed by the beam stop and were omitted from the refinement. Carbon-bound H atoms were placed in calculated positions (C—H = 0.95 Å for aromatic carbon atoms and C—H = 0.99 Å for methylene groups) and were included in the refinement in the riding model approximation, with *U*(H) set to 1.2*U*_eq_(C). The H atoms of the methyl groups were refined as rotationally disordered over two positions (orientations separated by 60° rotation of the H atoms) and allowed to rotate with a fixed angle around the C—C bond to best fit the experimental electron density and to account for rotational disorder (HFIX 127 in the *SHELX* program suite (Sheldrick, 2008)), with *U*(H) set to 1.5*U*_eq_(C). Occupancies refined to 0.68 (2) and 0.32 (2). The H atom of the hydroxy group was allowed to rotate with a fixed angle around the C—O bond to best fit the experimental electron density (HFIX 147 in the *SHELX* program suite (Sheldrick, 2008)), with *U*(H) set to 1.5*U*_eq_(O). The disorder of the hydroxy group was handled with a disorder model over two positions and site occupancy factors of 0.856 (2) and 0.144 (2). The anisotropic displacement parameters of the two oxygen atoms and of the two carbon atoms of the disordered group were each constrained to be identical. Equivalent bond distances in the disordered sections (C2—O1 and C2b—O1b, C1—C2 and C1—C2b, and C2—C3 and C2b—C3) were restrained to be the same within a standard deviation of 0.02 Å. Some minor residual electron density – that could not be resolved in any chemically meaningful way – remained.

### Crystal data

**Table:**

C_23_H_27_FN_4_O_3_	*F*(000) = 904
*M**_r_* = 426.49	*D*_x_ = 1.366 Mg m^−^^3^
Monoclinic, *P*2_1_/*c*	Melting point = 431–433 K
Hall symbol: -P 2ybc	Mo *K*α radiation, λ = 0.71073 Å
*a* = 6.8537 (1) Å	Cell parameters from 8433 reflections
*b* = 21.5613 (5) Å	θ = 2.4–28.3°
*c* = 15.3472 (3) Å	µ = 0.10 mm^−^^1^
β = 113.857 (1)°	*T* = 200 K
*V* = 2074.15 (7) Å^3^	Platelet, colourless
*Z* = 4	0.52 × 0.37 × 0.23 mm

### Data collection

**Table:**

Bruker APEXII CCD diffractometer	5138 independent reflections
Radiation source: fine-focus sealed tube	4130 reflections with *I* > 2σ(*I*)
graphite	*R*_int_ = 0.015
φ and ω scans	θ_max_ = 28.3°, θ_min_ = 1.9°
Absorption correction: multi-scan (*SADABS*; Bruker, 2008)	*h* = −9→5
*T*_min_ = 0.944, *T*_max_ = 1.000	*k* = −28→28
19267 measured reflections	*l* = −19→20

### Refinement

**Table:**

Refinement on *F*^2^	Primary atom site location: structure-invariant direct methods
Least-squares matrix: full	Secondary atom site location: difference Fourier map
*R*[*F*^2^ > 2σ(*F*^2^)] = 0.045	Hydrogen site location: inferred from neighbouring sites
*wR*(*F*^2^) = 0.130	H-atom parameters constrained
*S* = 1.07	*w* = 1/[σ^2^(*F*_o_^2^) + (0.0609*P*)^2^ + 0.7502*P*] where *P* = (*F*_o_^2^ + 2*F*_c_^2^)/3
5138 reflections	(Δ/σ)_max_ < 0.001
291 parameters	Δρ_max_ = 0.39 e Å^−^^3^
3 restraints	Δρ_min_ = −0.30 e Å^−^^3^

### Special details

**Table:**

Geometry. All esds (except the esd in the dihedral angle between two l.s. planes) are estimated using the full covariance matrix. The cell esds are taken into account individually in the estimation of esds in distances, angles and torsion angles; correlations between esds in cell parameters are only used when they are defined by crystal symmetry. An approximate (isotropic) treatment of cell esds is used for estimating esds involving l.s. planes.

### Fractional atomic coordinates and isotropic or equivalent isotropic displacement parameters (Å^2^)

**Table:**

	*x*	*y*	*z*	*U*_iso_*/*U*_eq_	Occ. (<1)
F1	0.9472 (2)	−0.23734 (5)	0.06766 (8)	0.0637 (3)	
C1	0.3065 (2)	0.34124 (6)	0.10562 (9)	0.0263 (3)	
C2	0.4057 (3)	0.38777 (13)	0.0575 (3)	0.0303 (5)	0.856 (2)
H2	0.5363	0.4076	0.1061	0.036*	0.856 (2)
O1	0.4566 (2)	0.35414 (7)	−0.00902 (10)	0.0441 (4)	0.856 (2)
H1	0.5676	0.3332	0.0197	0.066*	0.856 (2)
C2B	0.371 (2)	0.3800 (9)	0.0602 (19)	0.0303 (5)	0.144 (2)
H2B	0.3836	0.3531	0.0096	0.036*	0.144 (2)
O1B	0.5863 (13)	0.4016 (4)	0.1090 (6)	0.0441 (4)	0.144 (2)
H1B	0.6707	0.3714	0.1204	0.066*	0.144 (2)
O2	−0.19649 (16)	0.28148 (5)	0.07163 (8)	0.0385 (3)	
O3	0.98743 (17)	−0.07016 (5)	0.27657 (8)	0.0355 (2)	
N1	0.09124 (17)	0.33395 (5)	0.07005 (8)	0.0243 (2)	
N2	0.43993 (18)	0.30815 (6)	0.17546 (9)	0.0304 (3)	
N3	0.19463 (19)	0.10440 (5)	0.16460 (8)	0.0297 (3)	
N4	0.8250 (2)	−0.02973 (6)	0.27986 (10)	0.0358 (3)	
C3	0.2461 (2)	0.43659 (7)	0.00112 (11)	0.0352 (3)	
H3A	0.2185	0.4652	0.0453	0.042*	
H3B	0.3052	0.4611	−0.0371	0.042*	
C4	0.0400 (2)	0.40636 (7)	−0.06404 (10)	0.0343 (3)	
H4A	−0.0587	0.4382	−0.1050	0.041*	
H4B	0.0687	0.3760	−0.1058	0.041*	
C5	−0.0608 (2)	0.37407 (7)	−0.00587 (10)	0.0310 (3)	
H5A	−0.1195	0.4056	0.0238	0.037*	
H5B	−0.1811	0.3484	−0.0486	0.037*	
C6	−0.0004 (2)	0.28750 (6)	0.10498 (10)	0.0257 (3)	
C7	0.1457 (2)	0.24916 (6)	0.17907 (10)	0.0256 (3)	
C8	0.3594 (2)	0.26122 (6)	0.21256 (10)	0.0283 (3)	
C9	0.5251 (2)	0.22464 (8)	0.29070 (12)	0.0410 (4)	
H9A	0.6444	0.2519	0.3278	0.061*	0.68 (2)
H9B	0.5769	0.1909	0.2629	0.061*	0.68 (2)
H9C	0.4623	0.2073	0.3325	0.061*	0.68 (2)
H9D	0.4780	0.1815	0.2877	0.061*	0.32 (2)
H9E	0.5455	0.2425	0.3525	0.061*	0.32 (2)
H9F	0.6601	0.2261	0.2830	0.061*	0.32 (2)
C10	0.0456 (2)	0.19768 (6)	0.21276 (11)	0.0321 (3)	
H10A	−0.0903	0.2126	0.2135	0.038*	
H10B	0.1416	0.1864	0.2788	0.038*	
C11	0.0019 (2)	0.13990 (7)	0.14944 (12)	0.0365 (3)	
H11A	−0.1000	0.1128	0.1624	0.044*	
H11B	−0.0659	0.1528	0.0818	0.044*	
C21	0.1578 (3)	0.06087 (7)	0.08649 (12)	0.0398 (4)	
H21A	0.1067	0.0839	0.0255	0.048*	
H21B	0.0453	0.0310	0.0833	0.048*	
C22	0.3598 (3)	0.02553 (7)	0.09942 (11)	0.0370 (3)	
H22A	0.4695	0.0550	0.0983	0.044*	
H22B	0.3286	−0.0040	0.0461	0.044*	
C23	0.4457 (2)	−0.01007 (7)	0.19418 (11)	0.0325 (3)	
H23	0.3353	−0.0411	0.1920	0.039*	
C24	0.4728 (3)	0.03595 (7)	0.27396 (11)	0.0370 (3)	
H24A	0.5180	0.0134	0.3352	0.044*	
H24B	0.5860	0.0662	0.2794	0.044*	
C25	0.2667 (3)	0.07031 (7)	0.25493 (11)	0.0371 (3)	
H25A	0.1553	0.0403	0.2526	0.044*	
H25B	0.2889	0.0997	0.3076	0.044*	
C31	0.6466 (2)	−0.04472 (6)	0.20962 (10)	0.0299 (3)	
C32	0.6772 (2)	−0.09575 (6)	0.15598 (10)	0.0300 (3)	
C33	0.8930 (2)	−0.10890 (6)	0.20159 (10)	0.0296 (3)	
C34	0.9956 (3)	−0.15634 (7)	0.17502 (11)	0.0346 (3)	
H34	1.1440	−0.1646	0.2068	0.041*	
C35	0.8610 (3)	−0.19004 (7)	0.09821 (12)	0.0415 (4)	
C36	0.6432 (3)	−0.17991 (8)	0.04974 (12)	0.0480 (4)	
H36	0.5604	−0.2055	−0.0026	0.058*	
C37	0.5487 (3)	−0.13217 (7)	0.07852 (12)	0.0414 (4)	
H37	0.4001	−0.1242	0.0465	0.050*	

### Atomic displacement parameters (Å^2^)

**Table:**

	*U*^11^	*U*^22^	*U*^33^	*U*^12^	*U*^13^	*U*^23^
F1	0.0769 (8)	0.0536 (7)	0.0580 (7)	0.0184 (6)	0.0246 (6)	−0.0218 (5)
C1	0.0285 (7)	0.0250 (6)	0.0276 (6)	0.0007 (5)	0.0135 (5)	−0.0010 (5)
C2	0.0238 (10)	0.0304 (11)	0.0399 (8)	−0.0053 (8)	0.0161 (9)	0.0069 (7)
O1	0.0381 (7)	0.0552 (8)	0.0450 (8)	0.0164 (6)	0.0231 (6)	0.0159 (6)
C2B	0.0238 (10)	0.0304 (11)	0.0399 (8)	−0.0053 (8)	0.0161 (9)	0.0069 (7)
O1B	0.0381 (7)	0.0552 (8)	0.0450 (8)	0.0164 (6)	0.0231 (6)	0.0159 (6)
O2	0.0243 (5)	0.0380 (6)	0.0518 (7)	0.0046 (4)	0.0138 (5)	0.0109 (5)
O3	0.0336 (5)	0.0306 (5)	0.0390 (6)	0.0009 (4)	0.0111 (4)	−0.0088 (4)
N1	0.0258 (5)	0.0229 (5)	0.0244 (5)	0.0036 (4)	0.0105 (4)	0.0017 (4)
N2	0.0257 (6)	0.0310 (6)	0.0342 (6)	0.0010 (5)	0.0118 (5)	0.0038 (5)
N3	0.0293 (6)	0.0254 (5)	0.0320 (6)	0.0026 (4)	0.0099 (5)	0.0025 (5)
N4	0.0368 (7)	0.0295 (6)	0.0396 (7)	0.0040 (5)	0.0137 (6)	−0.0069 (5)
C3	0.0407 (8)	0.0291 (7)	0.0355 (8)	−0.0008 (6)	0.0153 (6)	0.0053 (6)
C4	0.0366 (8)	0.0336 (7)	0.0306 (7)	0.0041 (6)	0.0114 (6)	0.0057 (6)
C5	0.0289 (7)	0.0325 (7)	0.0286 (7)	0.0055 (5)	0.0085 (5)	0.0061 (5)
C6	0.0260 (6)	0.0237 (6)	0.0305 (6)	0.0032 (5)	0.0146 (5)	0.0000 (5)
C7	0.0275 (6)	0.0229 (6)	0.0297 (6)	0.0043 (5)	0.0151 (5)	0.0021 (5)
C8	0.0280 (7)	0.0271 (6)	0.0300 (7)	0.0037 (5)	0.0118 (5)	0.0020 (5)
C9	0.0302 (8)	0.0421 (9)	0.0441 (9)	0.0054 (6)	0.0082 (7)	0.0151 (7)
C10	0.0314 (7)	0.0280 (7)	0.0419 (8)	0.0047 (5)	0.0201 (6)	0.0080 (6)
C11	0.0264 (7)	0.0284 (7)	0.0512 (9)	−0.0004 (5)	0.0120 (6)	0.0038 (6)
C21	0.0369 (8)	0.0350 (8)	0.0362 (8)	0.0028 (6)	0.0031 (6)	−0.0050 (6)
C22	0.0392 (8)	0.0373 (8)	0.0293 (7)	0.0052 (6)	0.0086 (6)	−0.0032 (6)
C23	0.0336 (7)	0.0256 (6)	0.0385 (8)	0.0021 (5)	0.0148 (6)	0.0002 (5)
C24	0.0460 (9)	0.0348 (8)	0.0297 (7)	0.0147 (6)	0.0149 (6)	0.0054 (6)
C25	0.0453 (9)	0.0310 (7)	0.0407 (8)	0.0096 (6)	0.0233 (7)	0.0089 (6)
C31	0.0349 (7)	0.0238 (6)	0.0316 (7)	−0.0001 (5)	0.0141 (6)	0.0001 (5)
C32	0.0352 (7)	0.0249 (6)	0.0294 (7)	−0.0002 (5)	0.0125 (6)	−0.0011 (5)
C33	0.0367 (7)	0.0237 (6)	0.0292 (7)	−0.0020 (5)	0.0144 (6)	−0.0012 (5)
C34	0.0396 (8)	0.0301 (7)	0.0367 (8)	0.0040 (6)	0.0182 (6)	0.0000 (6)
C35	0.0566 (10)	0.0321 (8)	0.0377 (8)	0.0082 (7)	0.0211 (7)	−0.0054 (6)
C36	0.0575 (11)	0.0385 (9)	0.0383 (9)	0.0000 (8)	0.0093 (8)	−0.0139 (7)
C37	0.0396 (8)	0.0360 (8)	0.0385 (8)	−0.0001 (6)	0.0055 (7)	−0.0071 (6)

### Geometric parameters (Å, °)

**Table:**

F1—C35	1.3537 (18)	C9—H9A	0.9800
C1—C2B	1.277 (18)	C9—H9B	0.9800
C1—N2	1.3042 (18)	C9—H9C	0.9800
C1—N1	1.3596 (17)	C9—H9D	0.9800
C1—C2	1.556 (2)	C9—H9E	0.9800
C2—O1	1.406 (4)	C9—H9F	0.9800
C2—C3	1.512 (3)	C10—C11	1.533 (2)
C2—H2	1.0000	C10—H10A	0.9900
O1—H1	0.8400	C10—H10B	0.9900
C2B—O1B	1.434 (16)	C11—H11A	0.9900
C2B—C3	1.555 (15)	C11—H11B	0.9900
C2B—H2B	1.0000	C21—C22	1.521 (2)
O1B—H1B	0.8400	C21—H21A	0.9900
O2—C6	1.2363 (16)	C21—H21B	0.9900
O3—C33	1.3559 (16)	C22—C23	1.536 (2)
O3—N4	1.4309 (16)	C22—H22A	0.9900
N1—C6	1.3990 (17)	C22—H22B	0.9900
N1—C5	1.4865 (16)	C23—C31	1.499 (2)
N2—C8	1.3811 (18)	C23—C24	1.528 (2)
N3—C11	1.4615 (18)	C23—H23	1.0000
N3—C21	1.4619 (19)	C24—C25	1.515 (2)
N3—C25	1.4671 (18)	C24—H24A	0.9900
N4—C31	1.3019 (19)	C24—H24B	0.9900
C3—C4	1.509 (2)	C25—H25A	0.9900
C3—H3A	0.9900	C25—H25B	0.9900
C3—H3B	0.9900	C31—C32	1.4392 (19)
C4—C5	1.503 (2)	C32—C33	1.386 (2)
C4—H4A	0.9900	C32—C37	1.399 (2)
C4—H4B	0.9900	C33—C34	1.392 (2)
C5—H5A	0.9900	C34—C35	1.374 (2)
C5—H5B	0.9900	C34—H34	0.9500
C6—C7	1.4344 (18)	C35—C36	1.390 (3)
C7—C8	1.3667 (19)	C36—C37	1.381 (2)
C7—C10	1.5023 (19)	C36—H36	0.9500
C8—C9	1.4994 (19)	C37—H37	0.9500
			
C2B—C1—N2	121.6 (7)	H9D—C9—H9E	109.5
C2B—C1—N1	114.6 (7)	C8—C9—H9F	109.5
N2—C1—N1	123.57 (12)	H9A—C9—H9F	56.3
N2—C1—C2	116.57 (14)	H9B—C9—H9F	56.3
N1—C1—C2	119.70 (13)	H9C—C9—H9F	141.1
O1—C2—C3	106.2 (2)	H9D—C9—H9F	109.5
O1—C2—C1	107.2 (2)	H9E—C9—H9F	109.5
C3—C2—C1	111.50 (15)	C7—C10—C11	112.43 (12)
O1—C2—H2	110.6	C7—C10—H10A	109.1
C3—C2—H2	110.6	C11—C10—H10A	109.1
C1—C2—H2	110.6	C7—C10—H10B	109.1
C1—C2B—O1B	116.0 (15)	C11—C10—H10B	109.1
C1—C2B—C3	126.9 (14)	H10A—C10—H10B	107.9
O1B—C2B—C3	104.9 (11)	N3—C11—C10	113.10 (12)
C1—C2B—H2B	101.6	N3—C11—H11A	109.0
O1B—C2B—H2B	101.6	C10—C11—H11A	109.0
C3—C2B—H2B	101.6	N3—C11—H11B	109.0
C2B—O1B—H1B	109.5	C10—C11—H11B	109.0
C33—O3—N4	107.11 (10)	H11A—C11—H11B	107.8
C1—N1—C6	120.76 (11)	N3—C21—C22	111.62 (12)
C1—N1—C5	123.39 (11)	N3—C21—H21A	109.3
C6—N1—C5	115.84 (11)	C22—C21—H21A	109.3
C1—N2—C8	118.38 (12)	N3—C21—H21B	109.3
C11—N3—C21	111.13 (12)	C22—C21—H21B	109.3
C11—N3—C25	110.49 (12)	H21A—C21—H21B	108.0
C21—N3—C25	109.50 (12)	C21—C22—C23	110.76 (13)
C31—N4—O3	107.63 (11)	C21—C22—H22A	109.5
C4—C3—C2	110.17 (15)	C23—C22—H22A	109.5
C4—C3—C2B	101.3 (7)	C21—C22—H22B	109.5
C4—C3—H3A	109.6	C23—C22—H22B	109.5
C2—C3—H3A	109.6	H22A—C22—H22B	108.1
C2B—C3—H3A	107.6	C31—C23—C24	113.02 (12)
C4—C3—H3B	109.6	C31—C23—C22	112.03 (12)
C2—C3—H3B	109.6	C24—C23—C22	108.06 (12)
C2B—C3—H3B	120.2	C31—C23—H23	107.8
H3A—C3—H3B	108.1	C24—C23—H23	107.8
C5—C4—C3	109.78 (12)	C22—C23—H23	107.8
C5—C4—H4A	109.7	C25—C24—C23	111.19 (13)
C3—C4—H4A	109.7	C25—C24—H24A	109.4
C5—C4—H4B	109.7	C23—C24—H24A	109.4
C3—C4—H4B	109.7	C25—C24—H24B	109.4
H4A—C4—H4B	108.2	C23—C24—H24B	109.4
N1—C5—C4	112.96 (12)	H24A—C24—H24B	108.0
N1—C5—H5A	109.0	N3—C25—C24	111.00 (12)
C4—C5—H5A	109.0	N3—C25—H25A	109.4
N1—C5—H5B	109.0	C24—C25—H25A	109.4
C4—C5—H5B	109.0	N3—C25—H25B	109.4
H5A—C5—H5B	107.8	C24—C25—H25B	109.4
O2—C6—N1	119.85 (12)	H25A—C25—H25B	108.0
O2—C6—C7	124.13 (12)	N4—C31—C32	111.00 (13)
N1—C6—C7	116.02 (11)	N4—C31—C23	120.13 (13)
C8—C7—C6	119.12 (12)	C32—C31—C23	128.87 (13)
C8—C7—C10	125.46 (12)	C33—C32—C37	119.37 (13)
C6—C7—C10	115.42 (12)	C33—C32—C31	103.89 (12)
C7—C8—N2	122.05 (12)	C37—C32—C31	136.70 (14)
C7—C8—C9	123.40 (13)	O3—C33—C32	110.37 (12)
N2—C8—C9	114.54 (12)	O3—C33—C34	125.22 (13)
C8—C9—H9A	109.5	C32—C33—C34	124.39 (13)
C8—C9—H9B	109.5	C35—C34—C33	113.34 (14)
H9A—C9—H9B	109.5	C35—C34—H34	123.3
C8—C9—H9C	109.5	C33—C34—H34	123.3
H9A—C9—H9C	109.5	F1—C35—C34	117.38 (15)
H9B—C9—H9C	109.5	F1—C35—C36	117.24 (15)
C8—C9—H9D	109.5	C34—C35—C36	125.38 (14)
H9A—C9—H9D	141.1	C37—C36—C35	119.17 (15)
H9B—C9—H9D	56.3	C37—C36—H36	120.4
H9C—C9—H9D	56.3	C35—C36—H36	120.4
C8—C9—H9E	109.5	C36—C37—C32	118.34 (15)
H9A—C9—H9E	56.3	C36—C37—H37	120.8
H9B—C9—H9E	141.1	C32—C37—H37	120.8
H9C—C9—H9E	56.3		
			
C2B—C1—C2—O1	−86 (16)	C6—C7—C8—C9	179.06 (14)
N2—C1—C2—O1	82.2 (2)	C10—C7—C8—C9	−1.5 (2)
N1—C1—C2—O1	−93.42 (18)	C1—N2—C8—C7	−0.7 (2)
C2B—C1—C2—C3	30 (16)	C1—N2—C8—C9	178.41 (13)
N2—C1—C2—C3	−161.95 (19)	C8—C7—C10—C11	−95.84 (17)
N1—C1—C2—C3	22.4 (3)	C6—C7—C10—C11	83.63 (15)
N2—C1—C2B—O1B	−22 (3)	C21—N3—C11—C10	−164.00 (13)
N1—C1—C2B—O1B	163.6 (13)	C25—N3—C11—C10	74.24 (15)
C2—C1—C2B—O1B	−9(14)	C7—C10—C11—N3	75.21 (16)
N2—C1—C2B—C3	−158.1 (15)	C11—N3—C21—C22	177.66 (13)
N1—C1—C2B—C3	27 (3)	C25—N3—C21—C22	−60.00 (17)
C2—C1—C2B—C3	−146 (18)	N3—C21—C22—C23	58.12 (17)
C2B—C1—N1—C6	171.9 (15)	C21—C22—C23—C31	−179.23 (12)
N2—C1—N1—C6	−2.7 (2)	C21—C22—C23—C24	−54.09 (17)
C2—C1—N1—C6	172.62 (19)	C31—C23—C24—C25	179.45 (12)
C2B—C1—N1—C5	−9.0 (15)	C22—C23—C24—C25	54.90 (17)
N2—C1—N1—C5	176.46 (13)	C11—N3—C25—C24	−177.07 (12)
C2—C1—N1—C5	−8.2 (2)	C21—N3—C25—C24	60.21 (17)
C2B—C1—N2—C8	−171.1 (16)	C23—C24—C25—N3	−59.11 (17)
N1—C1—N2—C8	3.1 (2)	O3—N4—C31—C32	0.47 (16)
C2—C1—N2—C8	−172.37 (19)	O3—N4—C31—C23	−179.79 (12)
C33—O3—N4—C31	−0.26 (15)	C24—C23—C31—N4	−7.0 (2)
O1—C2—C3—C4	66.51 (17)	C22—C23—C31—N4	115.40 (16)
C1—C2—C3—C4	−50.0 (3)	C24—C23—C31—C32	172.73 (14)
O1—C2—C3—C2B	106 (7)	C22—C23—C31—C32	−64.91 (19)
C1—C2—C3—C2B	−11 (6)	N4—C31—C32—C33	−0.51 (16)
C1—C2B—C3—C4	−53 (3)	C23—C31—C32—C33	179.79 (14)
O1B—C2B—C3—C4	167.3 (14)	N4—C31—C32—C37	176.94 (18)
C1—C2B—C3—C2	165 (9)	C23—C31—C32—C37	−2.8 (3)
O1B—C2B—C3—C2	25 (5)	N4—O3—C33—C32	−0.07 (15)
C2—C3—C4—C5	64.6 (2)	N4—O3—C33—C34	−178.55 (13)
C2B—C3—C4—C5	57.3 (11)	C37—C32—C33—O3	−177.66 (13)
C1—N1—C5—C4	21.57 (18)	C31—C32—C33—O3	0.33 (16)
C6—N1—C5—C4	−159.23 (12)	C37—C32—C33—C34	0.8 (2)
C3—C4—C5—N1	−48.73 (16)	C31—C32—C33—C34	178.83 (13)
C1—N1—C6—O2	−179.85 (12)	O3—C33—C34—C35	177.88 (14)
C5—N1—C6—O2	0.92 (18)	C32—C33—C34—C35	−0.4 (2)
C1—N1—C6—C7	−0.04 (17)	C33—C34—C35—F1	−179.75 (14)
C5—N1—C6—C7	−179.26 (11)	C33—C34—C35—C36	−0.3 (2)
O2—C6—C7—C8	−178.03 (13)	F1—C35—C36—C37	179.98 (16)
N1—C6—C7—C8	2.17 (18)	C34—C35—C36—C37	0.5 (3)
O2—C6—C7—C10	2.5 (2)	C35—C36—C37—C32	−0.1 (3)
N1—C6—C7—C10	−177.33 (11)	C33—C32—C37—C36	−0.6 (2)
C6—C7—C8—N2	−1.9 (2)	C31—C32—C37—C36	−177.72 (17)
C10—C7—C8—N2	177.58 (13)		

### Hydrogen-bond geometry (Å, °)

**Table:**

*D*—H···*A*	*D*—H	H···*A*	*D*···*A*	*D*—H···*A*
O1—H1···O2^i^	0.84	1.86	2.6945 (16)	174.
O1B—H1B···O2^i^	0.84	2.39	3.153 (8)	152.
C4—H4A···N4^ii^	0.99	2.55	3.4830 (19)	157.

Symmetry codes: (i) *x*+1, *y*, *z*; (ii) *x*−1, −*y*+1/2, *z*−1/2.
